# Sedentary patterns and cardiometabolic risk factors in Mexican children and adolescents: analysis of longitudinal data

**DOI:** 10.1186/s12966-022-01375-0

**Published:** 2022-12-01

**Authors:** Abeer A. Aljahdali, Ana Baylin, Edward A. Ruiz-Narvaez, Hyungjin Myra Kim, Alejandra Cantoral, Martha M. Tellez-Rojo, Margaret Banker, Karen E. Peterson

**Affiliations:** 1 Department of Clinical Nutrition, King Abdulaziz University, Jeddah, Saudi Arabia; 2 Department of Nutritional Sciences, University of Michigan, Ann Arbor, MI USA; 3 Department of Epidemiology, University of Michigan, Ann Arbor, MI USA; 4 Center for Computing, Analytics and Research, University of Michigan, Ann Arbor, MI USA; 5 Department of Health, Iberoamerican University, Mexico City, Mexico; 6 Center for Nutrition and Health Research, National Institute of Public Health, Cuernavaca, Mexico; 7 Department of Biostatistics, University of Michigan, Ann Arbor, MI USA; 8 Department of Environmental Health Sciences, University of Michigan, Ann Arbor, MI USA; 9 University of Michigan, School of Public Health, Ann Arbor, MI 48109-2029 USA

**Keywords:** Physical activity, Sedentary behavior, Screen time, Bouts, Accelerometer, Cardiometabolic health, Children and adolescents, Longitudinal data, Repeated measures study design

## Abstract

**Background:**

Sedentary behavior is a modifiable risk factor for cardiometabolic health; however, the assessment of total sedentary time may not capture youth’s highly active and interrupted activity patterns. This study examined the associations between sedentary activity patterns and cardiometabolic risk factors among Mexican youth, who have a disproportionate burden of metabolic diseases, using a repeated measure design out of a longitudinal data.

**Methods:**

570 subjects in the Early Life Exposure in Mexico to ENvironmental Toxicants (ELEMENT) birth cohort, who were followed up to three-time points during adolescence, were included. Bout duration, and frequency and percentages of waking time spent in specific intensities of activity, were quantified using ActiGraph wGT3X-BT wrist accelerometers. Self-reported questionnaires were used to query the usual duration of different sedentary behaviors. Outcomes were fasting lipid profile, markers for glucose homeostasis, anthropometry, and blood pressure. Associations were modeled using linear mixed-effects models, and isotemporal substitution approach was additionally used to assess the effect of replacing objectively assessed sedentary activity with other activity intensities, adjusting for potential confounders.

**Results:**

Each hour of self-reported screen-based time was positively associated with diastolic blood pressure (mm Hg) [β = 0.30, 95% confidence interval (95% CI) = 0.10, 0.51], and an hour of other sedentary time was associated with log serum glucose (mg/dL) [β = 0.01, 95% CI = 0.004, 0.017]. Substitution models showed that replacing 5% of sedentary time with moderate to vigorous physical activity (MVPA) was associated with lower waist circumference (cm) [β = − 1.35, 95% CI = − 1.91, − 0.79] and log serum triglycerides (mg/dL) [β = − 0.11, 95% CI = − 0.18, − 0.03]. Substituting one uninterrupted sedentary bout with light activity was associated with lower insulin (μIU/mL) [β = − 0.06, 95% CI = − 0.10, − 0.02].

**Conclusions:**

Sedentary time was associated with cardiometabolic risk factors in Mexican youth in a context-specific manner. Replacing sedentary time with higher intensities was associated with improvements in some cardiometabolic markers.

**Supplementary Information:**

The online version contains supplementary material available at 10.1186/s12966-022-01375-0.

## Background

Sedentary behavior is defined as “any waking behavior characterized by an energy expenditure ≤1.5 metabolic equivalents (METs), while in a sitting, reclining or lying posture” [1]. On the other hand, physical inactivity is defined as “insufficient physical activity level to meet present physical activity recommendations” [1]. Sedentary behavior and physical inactivity are not identical concepts [2], and that meeting the physical activity recommendations is not a guarantee for not being sedentary [3]. Thus, they are independent modifiable cardiovascular disease (CVD) risk factors [4]. Promoting physical activity and reducing sedentary behavior across the lifespan are strategies for preventing CVD [4], which is consistent with the proposed cardiometabolic abnormalities management strategies among children and adolescents [5].

Children have distinct patterns in engaging and accumulating physical activity, characterized as being highly active and interrupted [6]. Assessment of total time spent in physical activity or sedentary behavior will not capture how sporadic patterns are associated with cardiometabolic health [7]. Therefore, there is a need to examine the activity patterns to refine current recommendations for children for combating diseases [7]. One way to address this need is to examine the activity accumulation via the assessment of activity bouts [7], defined as uninterrupted time performing an intensity-specific activity. Bout assessment enriches our understanding of the activity pattern beyond what total minutes of activity may convey [8]. Previous studies have examined the associations between bouts of activity and cardiometabolic health in children and adolescents in north America, Europe, UK, New Zealand, and Australia [7]; nevertheless, inconsistent evidence was reported, due to limited studies comprehensively assessing the entire spectrum of intensity levels, and cardiometabolic risk factors other than adiposity [7].

Previous studies compared the activity level across different races in the USA [9–13]. Despite similar total minutes of physical activity, Hispanic American adolescents have fewer minutes of moderate and vigorous activity (MVPA) relative to European Americans [10]. Also, a decrease in physical activity level and minutes of MVPA among Mexican Americans aged 6–11 years old was reported, while increasing trends were seen among non-Hispanic White youth [11]. On the contrary, other studies have shown that despite of higher daily minutes of sedentary activity among Mexican Americans compared to non-Hispanic White youth, Mexican Americans have higher minutes of MVPA [13], and a higher level of physical activity compared to other Hispanic/Latino counterparts [9]. The difference in the activity patterns might be a reason for the inconsistent associations between physical activity and cardiometabolic risk factors across races/ethnicities [10, 12]. However, evidence about actual differences across racial/ethnic groups is constrained by small sample sizes in previous studies [10], and findings derived from Hispanic youth in the USA [9–13] can not necessarily be generalized to the Hispanics outside the USA due to the regional and cultural context and available resources and assets.

Given concerns about sample size and generalizability of previous studies [9–13] and documented insulin resistance among normal weight Mexican youth [14], it is crucial to understand the contribution of activity patterns on cardiometabolic risk factors among children and adolescents in Mexico. Thus, the aim of the study was to assess the associations between repeated measures sedentary activity patterns and cardiometabolic risk factors among children and adolescents in a Mexico City birth cohort study. Specifically, we investigated the direct association between self-reported sedentary hours and cardiometabolic profile. Additionally, using the objective sedentary time, we analyzed the effect of replacing the percentage of awake sedentary time and sedentary bouts with higher physical activity intensities on our outcomes. We hypothesized that a more sedentary pattern would be associated with an impaired cardiometabolic profile, higher waist circumference, blood pressure, triglycerides (TG), impaired glucose homeostasis, and lower high density lipoprotein cholesterol (HDL-C).

## Methods

### Study population

The study population was composed of children and adolescents enrolled at the Early Life Exposures in Mexico to Environmental Toxicant (ELEMENT) cohort study in Mexico City, Mexico [15, 16]. A description of the ELEMENT birth cohorts has been published elsewhere [17]. Briefly, 1012 mother/child dyads from low- to moderate-income populations visiting prenatal clinics [18] were recruited between 1997 and 2004. At childbirth, mothers completed self-reported sociodemographic questionnaires. A subset of 670 mothers participated in a randomized controlled trial (RCT) of daily calcium supplementation (1200 mg) during their pregnancies until 1-year postpartum [16, 17]. The research team conducted multiple follow-up visits for the offspring and collected information on physical growth, maturation, diet, physical activity, and clinical biomarkers of cardiometabolic health. The number of offspring who have been followed-up was determined by the available funds and aims of each follow-up study.

Participants in the 2011 follow-up visit, herein called Time 1, were 250 children aged between 8 and 14 years [17], and a priority was given for children with available prenatal biological samples. Time 2 was the 2015 follow-up visit, and 554 children in the middle of pubertal transition aged 10–18 years were re-recruited [17]. The subjects enrolled at Time 1 were given a priority (~ 90% returned), and additional children from the original cohorts were enrolled. In 2018, 519 adolescents aged 12–21 completed the last follow-up visit, called Time 3 (~ 94% returned). From Time 1 to Time 3, a self-reported physical activity questionnaire assessment was collected, while the objective physical activity assessment, using accelerometry, was completed only at Time 2 and 3.

**Fig. 1:**
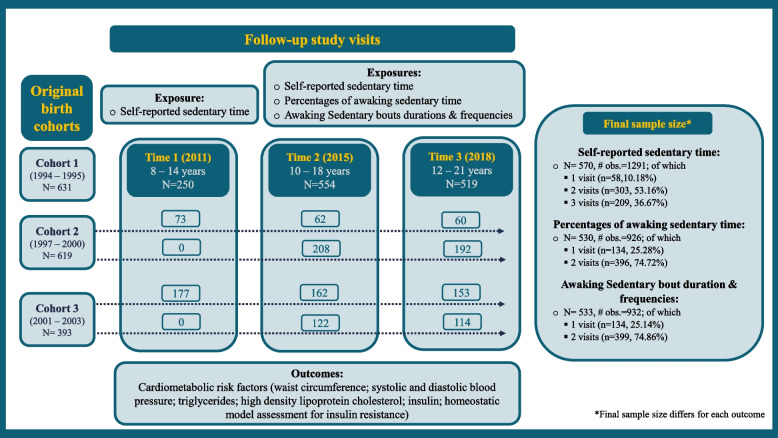
Flowchart of analytical samples of Early Life Exposures in Mexico to ENvironmental Toxicants (ELEMENT) cohort

The current sample size was 570 children and adolescents who attended at least one of the follow-up three visits and had information on sedentary behavior patterns and any cardiometabolic risk factors (waist circumference, systolic and diastolic blood pressure, fasting glucose, TG, HDL-C, insulin, and Homeostatic Model Assessment of Insulin Resistance (HOMA-IR)). Figure 1 illustrates the study design, sample size, and the number of repeated measures for each form of sedentary behavior assessment. The National Institute of Public Health of Mexico and the University of Michigan Institutional Review Boards approved the research protocols followed in the ELEMENT project. Upon the subjects’ enrollment in the project, the research team collected written informed consent and assent from mothers and adolescents, respectively.

### Cardiometabolic risk factors

#### Anthropometric measures

Trained research staff collected duplicate measurements for body weight (kilograms) to the nearest 0.1 kg and height (centimeters) to the nearest 0.5 cm using a digital scale (BAME Model 420; Catálogo Médico/ Tanita Co. Tokyo, Japan, with height rod (model WB-3000 m) [19], and waist circumference (centimeters) to the nearest 0.1 cm using a non-stretchable measuring tape (SECA (model 201, Hamburg, Germany)) [19]. The average of the two measurements was used for the analysis [20].

#### Cardiometabolic biomarkers

For Time 1 study visit, duplicate readings for systolic and diastolic blood pressure were recorded with participants in a seated position using Space Labs 90,217 Ambulatory Blood Pressure Measurement (Issaquah, WA, USA). Four cuff sizes: x-small (17–26 cm), small (24–32 cm), medium (32–42 cm), and large (38–50 cm), were available. For Time 2 and 3 study visits, duplicate readings for systolic and diastolic blood pressure were recorded with participants in a seated position using an automated blood pressure monitor (BPM-200 Medical Devices Blood Pressure Monitor, BpTRU; Coquitlam, BC, Canada). The following cuffs were available at these study visits: child cuff (13–18 cm), adult-small (18–26 cm), adult-regular (26–34 cm), adult-large (32–43 cm), and adult-extra-large (41–52 cm). Staff members assured the proper use of the cuff’s size based on the participant’s arm size. The average of the two blood pressure measurements was used for the analysis. Blood samples after fasting for ≥8 hours were used to analyze serum glucose via automated chemiluminescence immunoassay (Immulite®1000; Siemens Medical Solutions) [20], and TG and HDL-C using a biochemical analyzer (Cobas Mira Plus; Roche Diagnostics) [20]. Levels of insulin were quantified via enzyme-linked immunosorbent assay chemiluminescence method with Immulite® 1000, Erlangen, Germany equipment [19]. A Homeostatic Model Assessment of Insulin Resistance (HOMA-IR) was calculated as [fasting plasma glucose (mmol/L)*fasting serum insulin (mIU/mL))/ 22.5] [21]; higher values represent lower insulin sensitivity/insulin resistance [21].

### Physical activity and sedentary activity assessment

We assessed the physical activity and sedentary activity using two approaches: self-reported assessment for the sedentary activity and objective assessment for physical activity using the accelerometer. At each of the three follow-up visits, questionnaires modified from the Youth Activity Questionnaire (YAQ) and validated relative to 24 hours physical activity recall among Mexican school-children aged 10 to 14 years in Mexico City [22], were administered by research staff. The questionnaire queried the usual daily frequency of sedentary and select moderate-to-vigorous activities in the previous month. The questionnaires quantified the sedentary hours spent in the following activities: 1) hours spent watching TV (never, < 1, 1- < 2, 2–3, 4–5, 6–7, ≥ 8 hrs.), 2) hours spent watching movies or videos on a video cassette recorder (VCR) or digital versatile disc (DVD) (never, < 1, 1–2, 2–3, 4–5, 6–7, ≥ 8 hrs.), 3) hours spent doing homework or reading (never, 0.5, 0.5–1, 1–2, ≥ 3 hrs.), and 4) hours spent in commuting (i.e., riding a bus or car) (< 1, 1–2, 2–3, 3–4, ≥ 4 hrs.). Total metabolic equivalents (METs) per week were calculated by summing the METs for all physical activities in the questionnaire. METs for each activity were calculated by multiplying the corresponding METs based on Ainsworth’s et al. compendium [23] by activity intensity. The self-reported hours of sedentary activities used in this analysis were (1) daily total sedentary hours, which is a sum of the number of hours spent in all four types of sedentary activities, (2) daily screen-based sedentary hours calculated by combining the number of hours spent watching TV or movies, and (3) daily other sedentary hours was calculated by adding up the number of hours spent doing homework or reading and commuting.

During the last two follow-up visits, an objective physical activity assessment was obtained using the ActiGraph wGT3X-BT (ActiGraph LLC, Pensacola, FL). The water-resistant device [24] was worn on the non-dominant wrist for 24 hours for seven consecutive days, and a wristband was used to secure the ActiGraph snugly on the wrist. Children and adolescents who had accelerometer data from at least three weekdays and one weekend day [25, 26] were included in the analysis. A day with less than 10 hours of accelerometer data was counted as an invalid day and removed from the analysis [18]. The collected data were processed with ActiLife program (ActiGraph LLC. 2009, Version 6.13.3). Pruned dynamic programming separated the waking time from the sleeping time [27], and then we used the awake time data, which has been used in other studies [28, 29]. After that, actigraphy data were summarized into 5-second epochs, and Chandler’s Vector Magnitude (VM) cutoffs were used to classify the daily awake time into the following three categories of physical activity intensities: (1) sedentary [VM counts = 0–305], (2) light [VM counts = 306–817], (3) moderate and vigorous physical activity (MVPA) [VM counts = ≥818] [30]. Out of all available days per subject, the average total minutes per day of physical activities were calculated and then used to calculate the objective physical activity exposures. A bout was defined as 5 minutes of uninterrupted time performing a specific activity intensity. Within a bout, we allowed for up to 30-second of change in the physical activity intensity before terminating the bout.

The objective physical activity exposures assessed using the accelerometer were:

- The percentage of sedentary activity per day = (100*total minutes of sedentary activity during the awake time/total minutes of awake time).
- The percentage of light activity per day = (100*total minutes of light activity during the awake time /total minutes of awake time).
- The percentage of MVPA per day = (100*total minutes of MVPA during the awake time /total minutes of awake time).
- Bouts frequency (bouts/day) is the sum of all bouts that occurred per day for each physical activity intensity level
- Bouts duration (minutes/day) is the sum of bouts minutes occurred performing bouts throughout the day for each physical activity intensity level

### Potential confounders

Based on prior knowledge of cardiometabolic health, potential confounders included: 1) maternal and childbirth characteristics measured at baseline, e.g., sex, gestational age**,** mode of delivery, parity, mother’s age, marital status, years of education, and duration of breastfeeding. and 2) follow-up characteristics for the children, measured at each of the three visits, e.g., child’s age, body mass index (BMI), total daily caloric intake, and pubertal onset. We evaluated if the differences in original birth cohorts, assessed by mothers’ enrollment in the RCT, would be a potential confounding factor to account for.

After childbirth, mothers reported household and demographic information, including their ages, marital status (married or other – free union, single, separated, or divorced), parity status (< 1, ≥2), and years of education (continuous), gestational age in weeks (continuous) estimated by a registered nurse, and mode of delivery (vaginal or C-section childbirth), enrollment at the RCT for calcium supplementation (not enrolled or enrolled). The newborns were followed until 5 years of age, and information about self-reported breastfeeding duration (continuous) was quantified across the visits [31].

Total caloric intake was calculated from a semi-quantitative food frequency questionnaire at each study visit [32, 33]. Sexual maturation was coded based on trained pediatrician assessment for the breast, pubic hair, and boys’ genitalia [34] to assess Tanner stage (i.e., the range of values were 1 for pre-pubertal status up to 5 for fully mature status) [35, 36]. We classified pubertal onset as having a value greater than 1 for the Tanner Stage for pubic hair or genital development for boys and pubic hair or breast development in girls, respectively [37, 38].

### Statistical analysis

Self-reported exposure variables included daily total sedentary time, screen-based sedentary time, and other sedentary time. Objective assessment of exposures included the percentage of waking time spent in specific intensities of physical activity (i.e., sedentary, light, and MVPA), bout duration, and bout frequency of specific intensities of physical activity (i.e., sedentary, light, and MVPA). Outcome measures were 1) waist circumference (cm), 2) systolic and 3) diastolic blood pressure (mm Hg), 4) fasting glucose (mg/dL), 5) fasting TG (mg/dL), 6) fasting HDL-C (mg/dL), 7) fasting insulin (μIU/mL), and 8) HOMA-IR. We log-transformed the HDL-C, TG, insulin, and HOMA-IR variables to minimize skewedness of their distributions. We calculated descriptive statistics for the analytic sample, such as mean and standard deviation for continuous variables and frequency (proportions) for categorical demographic characteristics.

We examined the relationship between sedentary activity patterns with outcomes of interests using linear mixed-effects  models with a compound symmetry error structure for repeatedly measured data within each participant. We included all subjects with available data in each model, resulting in a variable number of repeated measures for each subject. Residuals of the final models were checked by assessing the mixed effects assumptions. Findings are presented as β and 95% confidence intervals (95% CI). The beta-coefficient multiplied by 100 for log-transformed outcomes can be interpreted approximately as a percentage of change in outcome for each one unit increase in the exposure variable [39].

For each exposure, the crude model included only a continuous variable of the exposure. In the fully adjusted model, following a parsimonious approach, we included covariates if they were associated with the exposure of interest among our study population. Age at each study visit was included in all models to capture the time difference between the study visits. Also, sex, pubertal onset, and METs (i.e., for self-reported exposures), and total awake time spent on all physical activity intensities (i.e., for objective exposures) were included in the adjusted models. We also adjusted for BMI to account for body size [40] in the waist circumference models. Models of each outcome excluded subjects who had missing information for any covariates included in the fully adjusted model.

We used isotemporal substitution models for objective sedentary behavior exposures [41, 42]. We included total minutes of awake time, which is the sum of minutes spent in sedentary, light, and MVPA, and the percentage of awake time spent for light physical activity and for MVPA. The beta coefficient of the percentage of light activity in these models is interpreted as the change in outcome for substituting a percentage of awake time spent in sedentary activity with a percentage of light activity while keeping the total minutes of activity per day constant. The beta-coefficient of MVPA should be interpreted similarly as substituting a percentage of MVPA for a percentage of awake sedentary activity. For the bout duration analysis of light activity bout, we included the bout duration of sedentary activity with the bout duration for light activity in the same model. Bout frequency analysis of light activity was done similarly. Beta coefficients of replacing the awake sedentary activity with light activity were calculated by taking the difference in the point estimates of light and sedentary activities, and standard errors were calculated as $\sqrt{variance\ \left(\beta 1\right)+ variance\left(\beta 2\right)-2\ast \textrm{covariance}\ \left(\beta 1,\beta 2\right)}$ [43] . For bout duration and frequency of MVPA, we followed the same approach.

To assure the robustness of our conclusion, we examined the influence of outlier values by running the models after excluding outliers for each outcome (i.e., ≤ first quartile – (1.5*interquartile range) or ≥ third quartile + (1.5*interquartile range)). We accounted for multiplicity by correcting *p* of < 0.00625 (0.05/8 [number of outcomes]) as a statistically significant association. We considered our four exposures to be independent from each other. SAS statistical software package, version 9.4, was used for all analyses (SAS Corp, Cary, NC, USA).

## Results

Figure 1 illustrates the study design, sample sizes, and the number of repeated measures used for exposure and outcome. The final sample size for the self-reported sedentary time was 570 subjects, with up to three repeated measurements per subject. For percentages of awake sedentary time and sedentary bouts duration and frequencies, 530 and 533 subjects, respectively, were included with up to two repeated measurements per subject. The duration of follow-up ranged from 0 (i.e., subjects enrolled at one study visit only) – 7 years (mean (SD) 3.1 years (1.9), and the mean accelerometer wear time was 6.97 days at each study visit. Table 1 shows the demographic characteristics of the study population by time point. The mean (SD) age of the sample was 10.3 (1.7) years, 14.5 (2.1) years, and 16.4 (2.1) years at Time 1, 2, and 3, respectively. Among cardiometabolic risk factors, the mean values for waist circumference, TG, insulin, and HOMA-IR rose across the three visits. Self-reported sedentary time was relatively stable across the three visits, while the objective assessment using an accelerometer showed increased sedentary activity and decreased MVPA activity in Time 3 relevant to values reported at Time 2 (Table 1). We explored the crude correlation between the self-reported sedentary time (hours/day) with the objective sedentary time (hours/awaking day), and found weak correlations at Time 2, and Time 3, respectively (Pearson’s correlation is [ρ] = 0.12, (*p* = 0.01), and *ρ* = 0.03, (*p* = 0.60)).

**Table 1:** Descriptive Statistics of the Early Life Exposures in Mexico to ENvironmental Toxicants (ELEMENT) Analytical Sample

	Time 1 *N* = 250	Time 2 *N* = 554	Time 3 *N* = 519
**Maternal characteristics (at time of child’s birth)**			
**Years of education,** (years)	11 (2.8) ^1^	10.9 (2.9) ^2^	11 (2.9) ^3^
**Age at childbirth,** (years)	26.8 (5.6) ^1^	26.4 (5.4)^3^	26.4 (5.4) ^4^
**Parity (**≥ 2)**,** %	156 (62.4) ^1^	340 (61.4) ^2^	319 (61.5) ^3^
**Marital Status **(married), %	178 (71.2) ^1^	390 (70.4) ^4^	363 (69.9) ^5^
**Enrolled in calcium supplement study,** %	95 (38.) ^1^	150 (27.1) ^2^	138 (26.6) ^3^
**Youth characteristics (at birth)**			
**Girls,** %	132 (52.8)	286 (51.62)	273 (52.6)^1^
**Gestational age,** (weeks)	38.9 (1.5) ^6^	38.8 (1.6) ^7^	38.8 (1.6) ^8^
**Mode of delivery **(vaginal delivery), %	144 (57.6) ^9^	352 (63.54) ^5^	329 (63.4) ^7^
**Breastfeeding duration**, (months)	8.1 (5.9) ^1^	8.0 (6.1) ^2^	8 (6) ^3^
**Youth characteristics (at follow-up visit)**			
**Age,** (years)	10.3 (1.7)	14.5 (2.1)	16.4 (2.1)
**Body mass index,** (kg/m^2^)	19.4 (3.6)	21.6 (4.1)	22.8 (4.5)
**Pubertal onset,** %	104 (41.6)	509 (91.88) ^10^	518 (99.8) ^1^
**Total caloric intake,** (kcal/day)	2627.3 (837.8)	2299.1 (922.4)	2124.5 (835.7)
**Youth cardiometabolic risk factors**			
**Waist circumference,** (cm)	70.7 (10.7)	79.6 (11.4)	85.5 (11.8) ^11^
**Systolic blood pressure,** (mm Hg)	102.7 (10.2)	98.7 (9.9)	101.5 (9.8) ^11^
**Diastolic blood pressure,** (mm Hg)	65.5 (7.3)	63 (6.9)	64.1 (7.2) ^11^
**Fasting glucose,** (mg/dL)	87 (9.4) ^11^	77.8 (7.3) ^12^	90.2 (8.4) ^13^
**Fasting TG,** (mg/dL)	87.5 (44.4) ^11^	104 (55.9) ^12^	105.5 (50.1) ^13^
**Fasting HDL-C,** (mg/dL)	58.7 (11.9) ^11^	43.1 (8.6) ^12^	44.7 (9) ^13^
**Fasting insulin,** (μIU/mL)	6.3 (11) ^14^	19.1 (11.8) ^12^	19.2 (12.6) ^15^
**HOMA-IR**	1.6 (3.5) ^14^	3.7 (2.3) ^12^	4.3 (2.9) ^15^
**Self-reported assessment**			
**Daily total sedentary activity,** (hours/day)	5.5 (1.9)	5.9 (2.3)	5.4 (2.1) ^11^
**Total metabolic equivalents,** (METs/week)	31.4 (19.8)	57.2 (39)	45 (35.2) ^11^
**Objective assessment of awake time**			
**Total time of physical activity,** (hours/day)	N/A	15.3 (0.9) ^16^	15.4 (1.1) ^17^
**Total time of sedentary activity,** (hours/day)	N/A	10 (1.2) ^16^	10.5 (1.3) ^17^
**% of total time spent in sedentary activity**	N/A	65.5 (6.7) ^16^	68.2 (6.7) ^17^
**Number of sedentary bouts,** (bout/day)	N/A	36.7 (9.8) ^16^	40.5 (9.4) ^17^
**Duration of sedentary bouts,** (minutes/day)	N/A	322.7 (104.7) ^16^	374.8 (109.8) ^17^
**Total time of light activity,** (hours/day)	N/A	3.9 (0.7) ^16^	3.8 (0.8) ^17^
**% of total time spent in light activity**	N/A	25.9 (4.3) ^16^	24.5 (4.7) ^17^
**Number of light bouts,** (bout/day)	N/A	0.6 (0.8) ^16^	0.9 (1) ^17^
**Duration of light bouts,** (minutes/day)	N/A	3.89 (5.5) ^16^	5.5 (7) ^17^
**Total time of MVPA activity,** (hours/day)	N/A	1.3 (0.5) ^16^	1.1 (0.4) ^17^
**% of total time spent in MVPA activity**	N/A	8.6 (3.1) ^16^	7.3 (2.7) ^17^
**Number of MVPA bouts,** (bout/day)	N/A	0.2 (0.5) ^16^	0.1 (0.3) ^17^
**Duration of MVPA bouts** (minutes/day)	N/A	1.3 (3.6) ^16^	0.9 (2.3) ^17^

Means (SD) or count (percentages) are presented for continuous or categorical variables, respectively

Number of missing values 1.*n* = 1, 2.n = 5, 3.*n* = 6, 4.*n* = 7, 5.*n* = 8, 6.*n* = 4, 7.*n* = 9, 8.*n* = 10, 9.*n* = 3, 10.*n* = 11, 11.*n* = 2, 12.*n* = 154, 13.*n* = 143, 14.*n* = 174, 15.*n* = 144, 16.*n* = 36, 17.*n* = 84

*Abbreviations*: *TG* Triglycerides, *HDL-C* High density lipoprotein cholesterol, *HOMA-IR* Homeostatic model assessment of insulin resistance, *METs* Metabolic equivalents, *MVPA* Moderate and vigorous physical activity

### Association between self-reported daily hours of sedentary time and cardiometabolic risk factors

The distributions of potential confounders were examined across quartiles of self-reported total sedentary time (i.e., daily hours) (Supplementary Table S1). Mothers’ enrollment in the calcium intervention study, parity, and mode of childbirth showed notable differences across the quartiles, and thus they were included in the fully adjusted models. In adjusted models, 1 h of screen-based sedentary time was positively associated with diastolic blood pressure (mm Hg) [β = 0.30, 95% CI = 0.10, 0.51], and 1 h spent in other sedentary activities (i.e., doing homework or reading and commuting) was associated with log-serum glucose (mg/dL) [β = 0.01, 95% CI = 0.004, 0.017] (corresponding to 1.06% increase in serum glucose) (Table 2). Sensitivity analyses are presented in Supplementary Table S2. Removing outliers had minor impact on the point estimates and did not change significance of the results (i.e., diastolic blood pressure (mm Hg) [β = 0.28 (mm Hg), 95% CI = 0.08, 0.48], and log-serum glucose (mg/dL) [β = 0.01, 95% CI = 0.004, 0.016]) (Supplementary Table S2).

**Table 2:** Linear Mixed Models between Self-reported Daily Hours of Sedentary Time and Cardiometabolic Risk Factors

	**Waist circumference (cm)**			**Systolic blood pressure (mm Hg)**			**Diastolic blood pressure (mm Hg)**			**Log glucose (mg/dL)**		
	*N = 570, # obs. = 1291*			*N = 570, # obs. = 1290*			*N = 570, # obs. = 1290*			*N = 432, # obs. = 1008*		
	▪ 1 visit (*n* = 58,10.18%)			▪ 1 visit (*n* = 58,10.18%)			▪ 1 visit (*n* = 58,10.18%)			▪ 1 visit (*n* = 53,12.27%)		
	▪ 2 visits (*n* = 303,53.16%)			▪ 2 visits (*n* = 304,53.33%)			▪ 2 visits (*n* = 304,53.33%)			▪ 2 visits (*n* = 182,42.13%)		
	▪ 3 visits (*n* = 209,36.67%)			▪ 3 visits (*n* = 208,36.49%)			▪ 3 visits (*n* = 208,36.49%)			▪ 3 visits (*n* = 197,45.60%)		
	All sedentary time	Screen-based	Other sedentary time	All sedentary time	Screen-based	Other sedentary time	All sedentary time	Screen-based	Other sedentary time	All sedentary time	Screen-based	Other sedentary time
	(hour /day)	(hour /day)	(hour /day)	(hour /day)	time (hour /day)	(hour /day)	(hour /day)	time (hour /day)	(hour /day)	(hour /day)	time (hour /day)	(hour /day)
**Crude models**^a^												
β	−0.14	− 0.87**	1.58**	0.04	0.13	− 0.19	0.15	0.28	− 0.17	− 0.0006	− 0.0044	0.0084
Back transformed β	–	–	–	–	–	–	–	–	–	− 0.06%	− 0.44%	0.84%
95% CI	(−0.39, 0.11)	(−1.17, − 0.58)	(1.13, 2.03)	(− 0.20, 0.27)	(− 0.15, 0.41)	(− 0.62, 0.24)	(− 0.02, 0.33)	(0.08, 0.49)	(− 0.49, 0.15)	(− 0.004, 0.0029)	(− 0.0085, −0.0003)	(0.0021, 0.0147)
**Adjusted models**^b,c^												
β	−0.06	−0.1	0.05	0.09	0.17	−0.12	0.16	0.3*	−0.19	−0.0002	− 0.0044	0.0105*
Back transformed β	–	–	–	–	–	–	–	–	–	−0.02%	−0.44%	1.06%
95% CI	(−0.14, 0.03)	(−0.204, − 0.002)	(−0.11 0.21)	(− 0.15, 0.32)	(− 0.11, 0.45)	(−0.57, 0.32)	(− 0.01, 0.33)	(0.1, 0.51)	(− 0.52, 0.15)	(− 0.0036, 0.0032)	(−0.0085, − 0.0004)	(0.004, 0.017)
	**Log TG (mg/dL)**			**log HDL-C (mg/dL)**			**Log insulin (μIU/mL)**			**Log HOMA-IR**		
	*N = 432, # obs. = 1008*			*N = 432, # obs. = 1008*			*N = 407, # obs. = 837*			*N = 407, # obs. = 837*		
	▪ 1 visit (*n* = 53,12.27%)			▪ 1 visit (*n* = 53,12.27%)			▪ 1 visit (*n* = 40,9.83%)			▪ 1 visit (*n* = 40,9.83%)		
	▪ 2 visits (*n* = 182,42.13%)			▪ 2 visits (*n* = 182,42.13%)			▪ 2 visits (*n* = 304,76.69%)			▪ 2 visits (*n* = 304,76.69%)		
	▪ 3 visits (*n* = 197,45.60%)			▪ 3 visits (*n* = 197,45.60%)			▪ 3 visits (*n* = 63,15.48%)			▪ 3 visits (*n* = 63,15.48%)		
	All sedentary time	Screen-based	Other sedentary time	All sedentary time	Screen-based	Other sedentary time	All sedentary time	Screen-based	Other sedentary time	All sedentary time	Screen-based	Other sedentary time
	(hour /day)	time (hour /day)	(hour /day)	(hour /day)	time (hour /day)	(hour /day)	(hour /day)	time (hour /day)	(hour /day)	(hour /day)	time (hour /day)	(hour /day)
**Crude models**^a^												
β	−0.0019	−0.0038	0.0024	−0.0031	0.0103	−0.0336**	0.0106	−0.0182	0.0765*	0.0098	−0.0238	0.0865*
Back transformed β	−0.19%	−0.38%	0.24%	−0.31%	1.04%	−3.30%	1.07%	−1.80%	7.95%	0.98%	−2.35%	9.04%
95% CI	(−0.0139, 0.0101)	(−0.0181, 0.0106)	(− 0.0192, 0.024)	(− 0.0096, 0.0034)	(0.0026, 0.018)	(− 0.045, − 0.0221)	(−0.015, 0.0363)	(− 0.0489, 0.0126)	(0.0302, 0.1228)	(− 0.0162, 0.0358)	(− 0.0549, 0.0074)	(0.0397, 0.1333)
**Adjusted models**^b^												
β	−0.0019	0.0045	−0.0178	−0.0018	− 0.0005	−0.0054	0.007	0.0063	0.0096	0.0073	0.0027	0.0192
Back transformed β	−0.19%	0.45%	−1.76%	−0.18%	− 0.05%	−0.54%	0.70%	0.63%	0.96%	0.73%	0.27%	1.94%
95% CI	(−0.0137, 0.0099)	(−0.0098, 0.0189)	(− 0.04, 0.0045)	(− 0.0071, 0.0035)	(−0.0069, 0.006)	(− 0.0153, 0.0046)	(− 0.0157, 0.0297)	(−0.0213, 0.0338)	(− 0.0326, 0.0518)	(− 0.0156, 0.0303)	(−0.0252, 0.0305)	(− 0.0234, 0.0617)

^a^Models includes either all sedentary time, screen-based sedentary time, or other sedentary time as a fixed effect and compound symmetry error matrix structure

^b^Models additionally adjusted for the following fixed effects: mother’s enrollment in the calcium intervention study, parity status, mode of childbirth at childbirth, child age, sex, metabolic equivalents, and pubertal onset

^c^Waist circumference models were additionally adjusted for body mass index

* *p* < 0.00625

** *p* < 0.0001

Back-transform β is expressed as a percentage of change

*Abbreviations*: *TG* Triglycerides, *HDL-C* High density lipoprotein cholesterol, *HOMA-IR* Homeostatic model assessment of insulin resistance

### Associations for substituting percentages of daily awake time spent on sedentary activity with higher intensities of physical activity on cardiometabolic risk factors

Quartiles of the percentage of MVPA showed slightly different distributions for mothers’ enrollment in the calcium intervention study, parity, mode of childbirth, sex, and pubertal status (Supplementary Table S3). The covariate-adjusted association was significant only for waist circumference and serum TG. In adjusted models, substituting 5% of an individual’s daily sedentary time out of the total minutes of awake time with MVPA was associated with a reduction in waist circumference by 1.35 cm (95% CI = (− 1.91, − 0.79) and a decrease in log-serum TG (mg/dL) by − 0.11 (95% CI = − 0.18, − 0.03), corresponding to 10% reduction in serum TG. (Table 3). Removing outlier values resulted in no notable difference in the point estimates for waist circumference (cm) and log serum TG (mg/dL) (Supplementary Table S4).

**Table 3:** Linear Mixed Models for Substituting Awake Sedentary Time with Higher-intensity Physical Activity and Cardiometabolic Risk Factors

			WC (cm)	SBP (mm Hg)	DBP (mm Hg)	Log glucose (mg/dL)	Log TG (mg/dL)	Log HDL-C (mg/dL)	Log insulin (μIU/mL)	Log HOMA-IR
			*N* = 530, # obs. = 926	*N* = 530, # obs. = 925	*N* = 530, # obs. = 925	*N* = 388, # obs. = 679	N = 388, # obs. = 679	*N* = 388, # obs. = 679	*N* = 388, # obs. = 679	*N* = 388, # obs. = 679
			▪ 1 visit (*n* = 134,25.28%)	▪ 1 visit (*n* = 135,25.47%)	▪ 1 visit (n = 135,25.47%)	▪ 1 visit (*n* = 97,25.00%)	▪ 1 visit (*n* = 97,25.00%)	▪ 1 visit (*n* = 97,25.00%)	▪ 1 visit (*n* = 97,25.00%)	▪ 1 visit (*n* = 97,25.00%)
			▪ 2 visits (*n* = 396,74.72%)	▪ 2 visits (*n* = 395,74.53%)	▪ 2 visits (*n* = 395,74.53%)	▪ 2 visits (*n* = 291,75.00%)	▪ 2 visits (*n* = 291,75.00%)	▪ 2 visits (*n* = 291,75.00%)	▪ 2 visits (*n* = 291,75.00%)	▪ 2 visits (*n* = 291,75.00%)
**Crude models**^a^										
5% of daily awake time spent on sedentary activity	Sedentary	Ref.								
	Light	β	1.26*	0.42	−0.72	−0.0002	0.0044	−0.0224	0.0358	0.0238
		Back transformed β	–	–	–	−0.02%	0.44%	−2.22%	3.64%	2.41%
		95% CI	(0.42, 2.10)	(−0.44, 1.29)	(−1.36, − 0.08)	(− 0.013, 0.0126)	(−0.04247, 0.0512)	(− 0.0415, − 0.0033)	(−0.0175, 0.0891)	(− 0.0335, 0.0811)
	MVPA	β	−5.42**	−2.47*	−0.49	−0.0026	− 0.1028*	0.0101	− 0.0765	−0.1168*
		Back transformed β	–	–	–	−0.26%	−9.77%	1.02%	−7.36%	−11.02%
		95% CI	(−6.72, −4.12)	(−3.81, − 1.13)	(− 1.48, 0.49)	(− 0.0392, − 0.002)	(− 0.1711, − 0.0346)	(−0.0178, 0.0380)	(− 0.1543, 0.0013)	(− 0.2004, − 0.0333)
**Adjusted models**^b,c^										
5% of daily awake time spent on sedentary activity	Sedentary	Ref.								
	Light	β	0.25	0.68	−0.54	0.0032	0.0006	−0.0196	0.0198	0.0189
		Back transformed β	–	–	–	0.32%	0.06%	−1.94%	2%	1.91%
		95% CI	(−0.1, 0.6)	(−0.155, 1.51)	(−1.16, 0.07)	(− 0.0099, 0.0163)	(− 0.0469, 0.0481)	(−0.0388, − 0.0004)	(−0.0338, 0.0733)	(− 0.0388, 0.0765)
	MVPA	β	1.35**	−0.86	0.42	−0.0083	−0.1053*	0.0195	−0.0873	− 0.0918
		Back transformed β	–	–	–	−0.83%	−9.99%	1.97%	−8.36%	−8.77%
		95% CI	(−1.91, −0.79)	(−2.19, 0.46)	(−0.56, 1.4)	(− 0.0275, 0.0108)	(− 0.1761, − 0.0345)	(−0.0094, 0.0485)	(− 0.1673, − 0.0073)	(−0.1778, − 0.0058)

^a^Models includes percentage of light and percentage of MVPA as fixed effects and compound symmetry error matrix structure

^b^Models additionally adjusted for the following fixed effects: mother’s enrollment in the calcium intervention study, mode of childbirth, parity status, child’s age, sex, total time of physical activity, and pubertal onset

^c^Waist circumference models were additionally adjusted for body mass index

* *p* < 0.00625

** *p* < 0.0001

Back-transform β is expressed as a percentage of change

*Abbreviations*: *WC* Waist circumference, *SBP* Systolic blood pressure, *DBP* Diastolic blood pressure, *TG* triglycerides, *HDL-C* High density lipoprotein cholesterol, *HOMA-IR* Homeostatic model assessment of insulin resistance

### Associations for substituting daily awake sedentary bout durations and frequencies with higher intensities of physical activities and cardiometabolic risk factors

Among the covariates, tertiles of MVPA bout frequency (Supplementary Table S5) and bout duration (Supplementary Table S6) were associated with mothers’ enrollment in the calcium intervention study, parity, sex, and pubertal status. The covariate-adjusted association was significant only for bouts of activity and serum insulin. Replacing one sedentary bout –defined as 5 min of uninterrupted time performing a specific intensity of activity– with one light activity bout was inversely associated with log-serum insulin (μIU/mL) [β = − 0.06, 95% CI = − 0.10, − 0.02] (i.e., 6% decrease). Moreover, substituting 1 min spent in sedentary bouts with 1 min of light activity bout was inversely associated with log-serum insulin (μIU/mL) [β = − 0.009, 95% CI = − 0.015, − 0.003] (i.e., 0.87% reduction) (Table 4). Removing outliers in log-serum insulin resulted in non-statistically significant associations at *p* < 0.00625 (Supplementary Table S7).

**Table 4:** Linear Mixed Models for Substituting Awake Sedentary Bouts^a^ with Higher-intensity Physical Activity and Cardiometabolic Risk Factors

			WC (cm) ^b^	SBP (mm Hg)	DBP (mm Hg)	Log glucose (mg/dL)	Log TG (mg/dL)	Log HDL-C (mg/dL)	Log insulin (μIU/mL)	Log HOMA-IR
			*N* = 533, # obs. = 932	*N* = 533, # obs. = 931	*N* = 533, # obs. = 931	*N* = 390, # obs. = 683	*N* = 390, # obs. = 683	*N* = 390, # obs. = 683	*N* = 390, # obs. = 683	*N* = 390, # obs. = 683
			▪ 1 visit (*n* = 134,25.14%)	▪ 1 visit (*n* = 135,25.33%)	▪ 1 visit (*n* = 135,25.33%)	▪ 1 visit (*n* = 97,24.87%)	▪ 1 visit (*n* = 97,24.87%)	▪ 1 visit (*n* = 97,24.87%)	▪ 1 visit (*n* = 97,24.87%)	▪ 1 visit (*n* = 97,24.87%)
			▪ 2 visits (*n* = 399,74.86%)	▪ 2 visits (*n* = 398,74.67%)	▪ 2 visits (*n* = 398,74.67%)	▪ 2 visits (*n* = 293,75.13%)	▪ 2 visits (*n* = 293,75.13%)	▪ 2 visits (*n* = 293,75.13%)	▪ 2 visits (*n* = 293,75.13%)	▪ 2 visits (*n* = 293,75.13%)
Substituting sedentary bout duration with light activity (minutes/ day)	Crude model^c^	β	0.07	0.17*	0.02	0.0021*	−0.0047	0.0008	−0.0108*	− 0.0078
		Back transformed β	–	–	–	0.21%	−0.47%	0.08%	−1.07%	−0.78%
		95% CI	(−0.02, 0.16)	(0.07, 0.26)	(−0.05, 0.09)	(0.0007, 0.0035)	(−0.0099, 0.0004)	(−0.0013, 0.0028)	(− 0.0165, − 0.0051)	(−0.014, − 0.0016)
	Adjusted model^c^	β	−0.01	0.05	−0.04	0.0012	−0.0035	0.0013	−0.0087*	−0.0075
		Back transformed β	–	–	–	0.12%	−0.35%	0.13%	−0.87%	−0.75%
		95% CI	(−0.05, 0.03)	(−0.05, 0.15)	(− 0.12, 0.03)	(− 0.0003, 0.0027)	(−0.0088, 0.0019)	(− 0.0008, 0.0034)	(− 0.0146, − 0.0028)	(−0.0139, − 0.0011)
Substituting sedentary bout duration with MVPA activity	Crude model^d^	β	−0.29*	−0.14	− 0.09	−0.0006	− 0.0097	0.0013	− 0.0107	−0.0125
(minutes/ day)		Back transformed β	–	–	–	−0.06%	−0.97%	0.13%	−1.06%	−1.24%
		95% CI	(−0.47, − 0.12)	(− 0.32, 0.05)	(−0.23, 0.05)	(− 0.0033, 0.0021)	(− 0.0189, − 0.0005)	(−0.0024, 0.005)	(− 0.0211, − 0.0003)	(−0.0238, − 0.0013)
	Adjusted model^d^	β	−0.07	− 0.12	−0.07	− 0.0005	−0.0079	0.0022	−0.0085	− 0.0089
		Back transformed β	–	–	–	−0.05%	−0.79%	0.22%	−0.85%	− 0.89%
		95% CI	(−0.1454, 0.0004)	(− 0.3, 0.05)	(− 0.21, 0.06)	(−0.0031, 0.0022)	(− 0.0171, 0.0013)	(− 0.0015, 0.0059)	(−0.0188, 0.0019)	(− 0.0201, 0.0022)
Substituting sedentary bout frequency with light activity (count/day)	Crude model^e^	β	0.5	1.18*	0.17	0.0145*	−0.0348	0.0061	−0.075*	−0.0541
		Back transformed β	–	–	–	0.01%	−3.42%	0.61%	−7.23%	−5.27%
		95% CI	(−0.14, 1.14)	(0.53, 1.83)	(−0.32, 0.65)	(0.0045, 0.0245)	(−0.0703, 0.0008)	(−0.0082, 0.0204)	(− 0.1147, − 0.0352)	(−0.0975, − 0.0108)
	Adjusted model^e^	β	−0.11	0.35	−0.32	0.0074	−0.0248	0.0081	−0.0585*	−0.0515
		Back transformed β	–	–	–	0.74%	−2.45%	0.81%	−5.68%	−5.02%
		95% CI	(−0.38, 0.16)	(−0.3, 1.01)	(− 0.81, 0.18)	(− 0.0031, 0.0180)	(−0.0618, 0.0122)	(− 0.0067, 0.0228)	(− 0.0996, − 0.0173)	(−0.0961, − 0.0069)
Substituting sedentary bout frequency with MVPA activity (count/day)	Crude model^f^	β	−2.12*	−1	−0.58	−0.0031	− 0.0717	0.0126	− 0.0604	−0.0736
		Back transformed β	–	–	–	−0.31%	−6.92%	1.27%	−5.86%	−7.10%
		95% CI	(−3.35, −0.9)	(−2.3, 0.31)	(−1.55, 0.39)	(−0.0215, 0.0153)	(− 0.1358, − 0.0076)	(−0.0134, 0.0386)	(− 0.1331, 0.0124)	(− 0.1521, 0.0048)
	Adjusted model^f^	β	−0.54	−0.88	− 0.44	−0.0034	− 0.0584	0.0203	− 0.0429	−0.0467
		Back transformed β	–	–	–	−0.34%	−5.67%	2.05%	−4.20%	−4.56%
		95% CI	(−1.05, −0.03)	(−2.13, 0.38)	(− 1.39, 0.51)	(− 0.0217, 0.0149)	(− 0.1228, 0.006)	(−0.0056, 0.0462)	(− 0.1152, 0.0295)	(− 0.1247, 0.0314)

^a^A bout was defined as 5 minutes of uninterrupted time performing a specific activity intensity. Within a bout, we allowed for up to 30-second of change in the physical activity intensity before terminating the bout

^b^Waist circumference models were additionally adjusted for body mass index

^c^Crude model includes bout duration spent in sedentary and light physical activity, and adjusted model was adjusted for mother’s enrollment in the calcium intervention study, parity status, child’s age, sex, total time of physical activity, and pubertal onset

^d^Crude model includes bout duration spent in sedentary and MVPA physical activity, and adjusted model was adjusted for mother’s enrollment in the calcium intervention study, parity status, child’s age, sex, total time of physical activity, and pubertal onset

^e^Crude model includes bout frequency spent in sedentary and light physical activity, and adjusted model was adjusted for mother’s enrollment in the calcium intervention study, parity status, child’s age, sex, total time of physical activity, and pubertal onset

^f^Crude model includes bout frequency spent in sedentary and MVPA physical activity, and adjusted model was adjusted for mother’s enrollment in the calcium intervention study, parity status, child’s age, sex, total time of physical activity, and pubertal onset

* *p* < 0.00625

** *p* < 0.0001

Back-transformed β is expressed as a percentage of change

*Abbreviations*: *WC* Waist circumference, *SBP* Systolic blood pressure, *DBP* Diastolic blood pressure, *TG* Triglycerides, *HDL-C* High density lipoprotein cholesterol, *HOMA-IR* Homeostatic model assessment of insulin resistance

## Discussion

To the best of the authors’ knowledge, the current study is the only prospective study with repeated measures of self-reported and objective sedentary patterns conducted among Mexican youth aged 8–21. Our sample had higher sedentary activity [44–47] but similar to Mexican Americans [9], a lower level of light activity [44, 45] but higher than Mexican Americans [9], and a higher level of MVPA [9, 44–48]. Although we found null associations between total self-reported sedentary hours, partitioning sedentary time by its context revealed that hours of screen time were associated with higher diastolic blood pressure. In addition, other sedentary time (i.e., doing homework or reading and commuting) was associated with higher serum glucose. Based on an objective assessment of sedentary time, substituting the percentage of sedentary time with MVPA was associated with a decrease in waist circumference and serum TG. Replacing sedentary bouts by light activity was associated with a reduction in serum insulin.

The lack of association between total sedentary time and cardiometabolic risk factors were consistent with some previous studies [8, 49–52], but contradicted other studies that found positive associations [10, 48, 49]. It is worth noting that multiple systematic reviews and meta-analyses of observational studies, including prospective and cross-sectional study designs, have found limited or lack of evidence of an association between sedentary time and cardiometabolic health among youth [44, 53–55]. Furthermore, evidence from a randomized cross-over study conducted among healthy youth supported the lack of any detrimental effects on cardiometabolic health after 8 h of uninterrupted sedentary activity [56]. Children and adolescents are metabolically healthy [46, 57] and a short exposure might not show noticeable impact compared to cumulative exposure over decades among middle-aged adult populations [57]. Despite the limited evidence for sedentary time among youth, several national public health authorities have incorporated the reduction of sedentary time in their physical activity guidelines [58, 59] as sedentary behavior is a modifiable risk factor for cardiovascular health across the lifespan [4].

We found a positive association between diastolic blood pressure and screen time. Our effect size was similar to one reported among adolescents aged 11–13 years in the US in a predominantly Hispanic population [50] . Other studies have detected detrimental associations between screen time and other cardiometabolic risk factors such as waist circumference, lipid profile, fat mass, and BMI [50, 60, 61] . Our positive association with blood pressure could be explained by prior evidence showing TV watching is associated with higher caloric consumption [62–64], impaired diet quality [62, 65], and shorter sleep duration [66], each of which is a plausible contributor to impaired cardiometabolic health. Nevertheless, three reviews concluded that there was little evidence from observational studies regarding the association between screen time and cardiometabolic health, including blood pressure, in youth [67–69] and flagged heterogeneity concerns across studies [67, 68].

A positive association between other sedentary time (i.e., doing homework or reading and commuting) and serum glucose was detected in our study. Previous experimental studies showed an increase in the mean ad libitum energy intake after cognitive-based sedentary tasks (i.e., reading and writing or computerized test-battery) relative to the control group (i.e., sitting in a comfortable chair) [70, 71]. Similarly, studies found that positive associations between mental work and caloric intake [72, 73], and between duration of stressful homework and total and trunk body fat percentages among boys [74]. Moreover, higher mean cortisol and larger variability in serum glucose and insulin while performing cognitive-based sedentary tasks have been reported [71]. This evidence suggests that cognitive-based sedentary time might contribute to positive energy balance and weight gain in the long-term [64, 70, 71, 75]; future studies are warranted to expand sedentary behavior assessment beyond the screen-time among youth.

Substituting sedentary time with MVPA models, showed inverse associations with waist circumference (β = − 1.35 cm) and log-serum TG (mg/Dl (β = − 0.11). Similarly, other studies have shown favorable associations for replacing sedentary time with MVPA on cardiometabolic health among youth [44, 45]. Thus, our results are consistent with the recommendations to replace sedentary time with activity at higher intensities to improve cardiometabolic health related outcomes among youth [53, 69, 76].

We found that replacing a sedentary bout with light activity was associated with a reduction in serum insulin. Studies have found inconsistent results of light activity on health outcomes [8, 46, 49, 77–79], with limited evidence from several reviews and meta-analyses [7, 53, 55, 69]. Some methodological related factors in defining bouts could be a source for the heterogeneity – as there is no consensus on defining the duration of a bout [8, 53, 66, 78]. In fact, there is a call for standardizing the exposure assessment to enhance evidence comparisons and hence the robustness of distilled evidence across studies [7, 45, 53, 55]. Moreover, our sample characteristics could be a reason for the reported small effect size; other studies showed larger effect sizes between sedentary and physical activity, and cardiometabolic health among subjects was overweight and obese [50, 77], and others explained that body fat percentages partially explained the association [80]. Thus, future studies are needed to examine if body composition modulates the association between activity and health outcomes.

Our study has several strengths. The use of a well-characterized cohort allowed for adjusting for multiple confounders at childbirth. Additionally, multiple limitations of the previous works were addressed through our longitudinal design with repeated measures. Using the repeated measures of activity acknowledges the change in activity patterns during growth and maturation [81, 82]. Different analytic perspectives were used; we examined the association of self-reported sedentary time as well as the substitution of sedentary behavior pattern with a higher intensity in relation to cardiometabolic risk factors. We also examined 24 hours of activity for seven consecutive days, as subjects wore the accelerometer continuously, as facilitated by the use of a water resistant device [27].

The study has several limitations, however. The sedentary time calculated from self-reported activity questionnaires has not been validated against an objective measure. Additionally, a few limitations resulted from our non-comprehensive assessment of all sedentary settings among youth. As a result, we might underestimate the sedentary hours in our study sample. We acknowledge the possibility of misclassification in our stratified sedentary time assessment, and future studies should apprise classifying the context of sedentary behaviors based on the available evidence. For the objective accelerometer data, we partially addressed the change in activity pattern across the weekend and weekday for school-age youth [83], by including subjects who had at least four valid days out of the 7 days, one of which had to be a weekend day. However, some researchers have claimed that 4 days may not fully represent variability in movement behaviors in youth [83], and could be a source of random error [84]. Moreover, to address the youth’s highly active and interrupted activity patterns [85], we summarized actigraphy data into 5-second epochs [30] to reduce the measurement error and the mis-classification associated with using longer epochs. However, there is no consensus about the epoch length used to summarize the accelerometer data; this is a concerning point as previous research showed the association between bouts of activity and metabolic health was influenced by the epoch length [8].

Despite the common use of accelerometers as a feasible objective assessment tool for activity in epidemiological studies [86–88], it is not a gold standard for assessing sedentary behavior [46]. Accelerometers do not distinguish between posture settings [1, 78, 85, 88], which could misclassify light activity (i.e., static standing) as sedentary time [78], or capture the context of sedentary behavior as they provide only a crude summary of total time of activity over the day [50, 88, 89]. Thus, an endorsement of assessing the sedentary behavior using two methods, whenever it is possible as they measure two dimensions of the same construct, was suggested [88]. Not all sedentary contexts are equal in their impacts on health due to their differences in caloric and food consumption [62–65, 70, 90], energy expenditure and biological homeostasis [71, 90] and other differences [66, 91, 92].

We considered a conservative alpha level to correct for the multiple testing, but we note that our results could still be due to chance. In addition, we could not rule out the possibility of residual confounding due to the use of crude assessment of some covariates and unknown confounding, such as family history of chronic diseases. Because the objective physical activity assessment was conducted after blood collection, this could be a potential source for reverse causation in our analysis. We acknowledge that the detected changes might not be of a clinical or public health significance given the small effect size; however, a greater sedentary time would result in larger effect sizes. Lastly, due to the existence of regional and cultural factors, our conclusions may not be generalizable to youth with Mexican heritage who do not live in Mexico City.

## Conclusions

In conclusion, we reported negative associations of screen-time and other sedentary time (i.e., doing homework or reading and commuting) and protective associations of replacing sedentary time by higher intensities on a few cardiometabolic risk factors among Mexican youth. Further studies are needed to consolidate the evidence around assessing sedentary and physical activity patterns using accelerometers. Currently, there is no consensus about the best approach to summarize accelerometer data, epoch length, and defining bouts, which is needed to enhance the comparability of research findings across studies, and reduce measurement error, and misclassifying duration of activity at different intensities [8, 50, 53, 66, 78]. For the sedentary time assessment, validation studies are needed to improve quality of self-reported sedentary time questionnaires against the objective assessment, which will allow comparing and complementing the evidence extracted from the two approaches. Additionally, we call for the use of objective assessment tools that can capture the context of the sedentary behavior. Furthermore, future studies are warranted to examine the context of sedentary behavior in relation to health outcomes to facilitate the incorporation of context-specific sedentary behavior recommendations among youth.

## Supplementary Information

**Additional file 1: Supplementary Table S1.** Overall Associations between Potential Confounders and Total Sedentary Time. **Supplementary Table S2.** Linear Mixed Models between Self-reported Daily Hours of Sedentary Time and Cardiometabolic Risk Factors. **Supplementary Table S3.** Overall Associations between Potential Confounders and Percentage of Moderate to Vigorous Physical Activity (MVPA). **Supplementary Table S4.** Linear Mixed Models for Substituting Awake Sedentary Time with Higher-intensity Physical Activity and Cardiometabolic Risk Factors. **Supplementary Table S5.** Overall Associations between Potential Confounders and Bout Frequency for Moderate to Vigorous Physical Activity (MVPA). **Supplementary Table S6.** Overall Associations between Potential Confounders and Bout Duration for Moderate to Vigorous Physical Activity (MVPA). **Supplementary Table S7.** Linear Mixed Models for Substituting Awake Sedentary Bouts^1^ with Higher-intensity Physical Activity and Cardiometabolic Risk Factors.

## Data Availability

The datasets supporting the conclusions of this article are not publicly available due to human subjects’ protections. The de-identified data are available upon reasonable request to corresponding author, Karen E. Peterson (karenep@umich.edu) following review and approval by the ELEMENT Executive Committee.

## Abbreviations

BMI: Body mass index
PA: Physical activity
WC: Waist circumference
SBP: Systolic blood pressure
DBP: Diastolic blood pressure
TG: Triglycerides
HDL-C: High density lipoprotein cholesterol
HOMA-IR: Homeostatic model assessment for insulin resistance
MVPA: Moderate and vigorous physical activity
METs: Metabolic equivalents
