# Supplementary material for: Sedentary patterns and cardiometabolic risk factors in Mexican children and adolescents: analysis of longitudinal data
Source: Int J Behav Nutr Phys Act. 2022 Dec 1;19:143. doi: 10.1186/s12966-022-01375-0 (PMC9714228; doi:10.1186/s12966-022-01375-0)
Supplement: Supplementary file 1 — Additional file 1: Supplementary Table S1. Overall Associations between Potential Confounders and Total Sedentary Time. Supplementary Table S2. Linear Mixed Models between Self-reported Daily Hours of Sedentary Time and Cardiometabolic Risk Factors. Supplementary Table S3. Overall Associations between Potential Confounders and Percentage of Moderate to Vigorous Physical Activity (MVPA). Supplementary Table S4. Linear Mixed Models for Substituting Awake Sedentary Time with Higher-intensity Physical Activity and Cardiometabolic Risk Factors. Supplementary Table S5. Overall Associations between Potential Confounders and Bout Frequency for Moderate to Vigorous Physical Activity (MVPA). Supplementary Table S6. Overall Associations between Potential Confounders and Bout Duration for Moderate to Vigorous Physical Activity (MVPA). Supplementary Table S7. Linear Mixed Models for Substituting Awake Sedentary Bouts1 with Higher-intensity Physical Activity and Cardiometabolic Risk Factors. [file 12966_2022_1375_MOESM1_ESM.docx]

**Supplementary Table S1:** Overall Associations between Potential Confounders and Total Sedentary Time:

|  | **Total Sedentary Time** | | | |
| --- | --- | --- | --- | --- |
|  | **Quartile 1**  Median= 3.25  n=328 | **Quartile 2**  Median= 4.63  n=331 | **Quartile 3**  Median=6.00  n=331 | **Quartile 4**  Median= 8.00  n=331 |
| **Maternal characteristics (at time of child’s birth)** | | | | |
| Years of education, (years) | 10.73 | 10.95 | 11.06 | 11.13 |
| Age at childbirth, (years) | 26.76 | 26.63 | 26.20 | 26.25 |
| **Parity (≥ 2), (%)** | 65.55 | 64.35 | 60.42 | 56.19 |
| Marital Status (married), (%) | 69.21 | 73.11 | 68.58 | 71.00 |
| **Enrolled in calcium supplement study, (%)** | 28.96 | 32.63 | 28.40 | 25.98 |
| **Youth characteristics (at birth)** | | | | |
| Girls, (%) | 49.70 | 49.55 | 54.08 | 55.59 |
| Gestational age, (weeks) | 38.92 | 38.78 | 38.75 | 38.66 |
| **Mode of delivery (vaginal delivery), (%)** | 64.63 | 65.26 | 60.73 | 58.91 |
| Breastfeeding duration, (months) | 7.63 | 8.32 | 8.15 | 8.07 |
| **Youth characteristics (at follow-up visit)** | | | | |
| Age, (years) | 14.50 | 14.37 | 14.25 | 14.74 |
| Body mass index, (kg/m^2^) | 21.58 | 21.66 | 21.49 | 21.90 |
| Pubertal onset, (%) | 85.98 | 83.69 | 84.89 | 87.61 |
| Total caloric intake, (kcal/day) | 2194.93 | 2272.28 | 2322.66 | 2377.76 |
| Metabolic equivalents, (METs/week) | 45.92 | 46.23 | 47.33 | 50.65 |

Means or percentages are presented for continuous or categorical variables, respectively

Bolded covariates included in the fully adjusted models for total sedentary time, screen-based sedentary time, and other sedentary time.

**Supplementary Table S2:** Linear Mixed Models between Self-reported Daily Hours of Sedentary Time and Cardiometabolic Risk Factors:

|  | **Waist circumference (cm)**  N= 568, # obs.= 1277 | | | **Systolic blood pressure (mm Hg)**  N= 570, # obs.= 1289 | | | **Diastolic blood pressure (mm Hg)**  N= 570, # obs.= 1286 | | | **Log glucose (mg/dL)**  N= 430, # obs.= 993 | | |
| --- | --- | --- | --- | --- | --- | --- | --- | --- | --- | --- | --- | --- |
|  | All sedentary time  (hour /day) | Screen-based  time (hour /day) | Other sedentary time (hour /day) | All sedentary time  (hour /day) | Screen-based  time (hour /day) | Other sedentary time (hour /day) | All sedentary time  (hour /day) | Screen-based  time (hour /day) | Other sedentary time (hour /day) | All sedentary time  (hour /day) | Screen-based  time (hour /day) | Other sedentary time (hour /day) |
| **Crude models ^1^** | | | | | | | | | | | | |
| β | -0.12 | -0.84** | 1.57** | 0.04 | 0.13 | -0.19 | 0.12 | 0.26 | -0.21 | -0.0009 | -0.0049 | 0.0086* |
| 95% CI | (-0.37, 0.13) | (-1.13, -0.55) | (1.13, 2.01) | (-0.20, 0.27) | (-0.15, 0.41) | (-0.62, 0.24) | (-0.05, 0.29) | (0.06, 0.46) | (-0.52, 0.11) | (-0.0041, 0.0022) | (-0.0087,  -0.0012) | (0.0028, 0.0143) |
| **Adjusted models ^2,3^** | | | | | | | | | | | | |
| β | -0.04 | -0.11 | 0.14 | 0.09 | 0.17 | -0.13 | 0.14 | 0.28* | -0.22 | -0.0006 | -0.0048 | 0.0099* |
| 95% CI | (-0.19, 0.12) | (-0.31, 0.08) | (-0.16, 0.44) | (-0.15, 0.32) | (-0.10, 0.45) | (-0.58, 0.31) | (-0.03, 0.31) | (0.08, 0.48) | (-0.55, 0.10) | (-0.0037, 0.0024) | (-0.0085,  -0.0011) | (0.0040, 0.0157) |
|  | **Log TG (mg/dL)**  N= 432, # obs.= 1002 | | | **Log HDL-C (mg/dL)**  N= 432, # obs.= 1004 | | | **Log insulin (μIU/mL)**  N= 400 , # obs.= 777 | | | **Log HOMA-IR**  N= 402 , # obs.= 786 | | |
|  | All sedentary time  (hour /day) | Screen-based  time (hour /day) | Other sedentary time (hour /day) | All sedentary time  (hour /day) | Screen-based  time (hour /day) | Other sedentary time (hour /day) | All sedentary time  (hour /day) | Screen-based  time (hour /day) | Other sedentary time (hour /day) | All sedentary time  (hour /day) | Screen-based  time (hour /day) | Other sedentary time (hour /day) |
| **Crude models ^1^** | | | | | | | | | | | | |
| β | -0.0022 | -0.0058 | 0.0061 | -0.0032 | 0.0101 | -0.0333** | 0.0161 | 0.0167 | 0.0155 | 0.0092 | -0.0011 | 0.0325 |
| 95% CI | (-0.0140, 0.0097) | (-0.0200, 0.0083) | (-0.0152, 0.0274) | (-0.0096, 0.0033) | (0.0024, 0.0177) | (-0.0447,  -0.0219) | (0.0006, 0.0317) | (-0.0022, 0.0356) | (-0.0127, 0.0436) | (-0.0084, 0.0268) | (-0.0225, 0.0204) | (0.0007, 0.0643) |
| **Adjusted models ^2^** | | | | | | | | | | | | |
| β | -0.0020 | 0.0025 | -0.0131 | -0.0019 | -0.0004 | -0.0058 | 0.0159 | 0.0216 | 0.0051 | 0.0091 | 0.0098 | 0.0090 |
| 95% CI | (-0.0136, 0.0096) | (-0.0117, 0.0166) | (-0.0351, 0.0089) | (-0.0072, 0.0033) | (-0.0068, 0.0060) | (-0.0159, 0.0040) | (0.0010, 0.0307) | (0.0034, 0.0398) | (-0.0224, 0.0325) | (-0.0071, 0.0253) | (-0.0101, 0.0297) | (-0.0208, 0.0389) |

^1^ Model includes either all sedentary time, screen-based sedentary time, or other sedentary time as a fixed effect and compound symmetry error matrix structure

^2^ Models additionally adjusted for the following fixed effects: mother’s enrollment in the calcium intervention study, parity status, mode of childbirth at childbirth, child age, sex, metabolic equivalents, and pubertal onset.

^3^ Waist circumference models were additionally adjusted for body mass index

* p < 0.00625

** p <0.0001

Abbreviations: TG: triglycerides; HDL-C: high density lipoprotein cholesterol; HOMA-IR: homeostatic model assessment of insulin resistance

**Supplementary Table S3:** Overall Associations between Potential Confounders and Percentage of Moderate to Vigorous Physical Activity (MVPA):

|  | **% of MVPA** | | | |
| --- | --- | --- | --- | --- |
|  | **Quartile 1**  Median= 4.77  n=238 | **Quartile 2**  Median= 6.81  n=238 | **Quartile 3**  Median= 8.62  n=238 | **Quartile 4**  Median= 11.52  n=238 |
| **Maternal characteristics (at time of child’s birth)** | | | | |
| Years of education, (years) | 11.18 | 11.05 | 10.96 | 10.71 |
| Age at childbirth, (years) | 26.76 | 26.47 | 26.72 | 25.62 |
| **Parity (≥ 2), (%)** | 64.71 | 57.98 | 59.41 | 61.34 |
| Marital Status (married), (%) | 73.95 | 72.69 | 66.95 | 67.65 |
| **Enrolled in calcium supplement study, (%)** | 22.69 | 27.73 | 29.29 | 31.09 |
| **Youth characteristics (at birth)** | | | | |
| **Girls, (%)** | 44.12 | 48.74 | 59.83 | 57.98 |
| Gestational age, (weeks) | 38.72 | 38.76 | 38.76 | 38.83 |
| **Mode of delivery (vaginal delivery), (%)** | 66.39 | 64.29 | 59.83 | 58.82 |
| Breastfeeding duration, (months) | 8.76 | 8.20 | 8.17 | 7.23 |
| **Youth characteristics (at follow-up visit)** | | | | |
| Age, (years) | 16.22 | 15.47 | 15.18 | 14.37 |
| Body mass index, (kg/m^2^) | 22.36 | 21.91 | 22.42 | 21.92 |
| **Pubertal onset, (%)** | 97.48 | 97.90 | 97.90 | 91.18 |
| Total caloric intake, (kcal/day) | 2181.56 | 2217.18 | 2164.57 | 2345.59 |
| Total minutes of activity, (minutes/day) | 925.28 | 917.24 | 922.96 | 911.38 |

Means or percentages are presented for continuous or categorical variables, respectively

Bolded covariates included in the fully adjusted substituting models for the percentage of sedentary activity.

**Supplementary Table S4:** Linear Mixed Models for Substituting Awake Sedentary Time with Higher Intensities and Cardiometabolic Risk Factors:

|  | | | **WC (cm)**  N= 527, # obs.= 918 | **SBP (mm Hg)**  N= 530, # obs.= 925 | **DBP (mm Hg)**  N= 529, # obs.= 919 | **Log glucose (mg/dL)**  N= 387, # obs.= 674 | **Log TG (mg/dL)**  N= 385, # obs.= 672 | **Log HDL-C (mg/dL)**  N= 384, # obs.= 670 | **Log insulin (μIU/mL)**  N= 384, # obs.= 669 | **Log HOMA-IR**  N= 385, # obs.= 669 |
| --- | --- | --- | --- | --- | --- | --- | --- | --- | --- | --- |
| **Crude models ^1^** | | | | | | | | | | |
| 5 % of daily awake time spent on sedentary activity | Sedentary | Ref. | | | | | | | | |
|  | Light | β | 1.37* | 0.41 | -0.71 | -0.0031 | 0.0049 | -0.0254* | 0.0470 | 0.0424 |
|  |  | 95 % CI | (0.54, 2.20) | (-0.45, 1.27) | (-1.32, -0.11) | (-0.0150, 0.0087) | (-0.0404, 0.0502) | (-0.0435, -0.0074) | (-0.0027, 0.0968) | (-0.0117, 0.0965) |
|  | MVPA | β | -5.38** | -2.41* | -0.52 | -0.0166 | -0.1074* | 0.0138 | -0.0744 | -0.1248* |
|  |  | 95 % CI | (-6.65, -4.11) | (-3.73, -1.09) | (-1.45, 0.42) | (-0.0337, 0.0005) | (-0.1730, -0.0417) | (-0.0124, 0.0400) | (-0.1463, -0.0026) | (-0.2028, -0.0468) |
| **Adjusted models ^2,3^** | | | | | | | | | | |
| 5 % of daily awake time spent on sedentary activity | Sedentary | Ref. | | | | | | | | |
|  | Light | β | 0.27 | 0.68 | -0.53 | 0.0013 | 0.0036 | -0.0224 | 0.0324 | 0.0372 |
|  |  | 95 % CI | (-0.08, 0.62) | (-0.14, 1.50) | (-1.12, 0.06) | (-0.0108, 0.0133) | (-0.0425, 0.0497) | (-0.0407, -0.0041) | (-0.0178, 0.0826) | (-0.0175, 0.0919) |
|  | MVPA | β | -1.33** | -0.87 | 0.33 | -0.0064 | -0.1110* | 0.0234 | -0.0850 | -0.1031 |
|  |  | 95 % CI | (-1.89, -0.77) | (-2.18, 0.44) | (-0.60, 1.26) | (-0.0239, 0.0111) | (-0.1791, -0.0428) | (-0.0039, 0.0506) | (-0.1590, -0.0109) | (-0.1834, -0.0227) |

^1^ Model includes the percentage of light and MVPA as fixed effects and compound symmetry error matrix structure.

^2^ Models additionally adjusted for the following fixed effects: mother’s enrollment in the calcium intervention study, mode of childbirth, parity status, child’s age, sex, total time of physical activity, and pubertal onset

^3^ Waist circumference models were additionally adjusted for body mass index

* p < 0.00625

** p <0.0001

Abbreviations: WC: waist circumference; SBP: systolic blood pressure; DBP: diastolic blood pressure; TG: triglycerides; HDL-C: high density lipoprotein cholesterol; HOMA-IR: homeostatic model assessment of insulin resistance

**Supplementary Table S5:** Overall Associations between Potential Confounders and Bout Frequency for Moderate to Vigorous Physical Activity (MVPA):

|  | **Bout frequency of MVPA** | | |
| --- | --- | --- | --- |
|  | **Tertile 1**  Median= 0  n=663 | **Tertile 2**  Median= 0.17  n=178 | **Tertile 3**  Median= 0.71  n=112 |
| **Maternal characteristics (at time of child’s birth)** | | | |
| Years of education, (years) | 10.91 | 10.92 | 11.48 |
| Age at childbirth, (years) | 26.49 | 25.94 | 26.55 |
| **Parity (≥ 2), (%)** | 61.54 | 61.80 | 55.36 |
| Marital Status (married), (%) | 70.44 | 69.10 | 71.43 |
| **Enrolled in calcium supplement study,** (%) | 28.21 | 23.60 | 31.25 |
| **Youth characteristics (at birth)** | | | |
| **Girls, (%)** | 54.75 | 53.37 | 39.29 |
| Gestational age, (weeks) | 38.76 | 38.63 | 39.01 |
| Mode of delivery (vaginal delivery), (%) | 62.44 | 60.67 | 64.29 |
| Breastfeeding duration, (months) | 8.35 | 7.19 | 7.97 |
| Age, (years) | 15.43 | 15.20 | 14.74 |
| **Youth characteristics (at follow-up visit)** | | | |
| Body mass index, (kg/m^2^) | 22.37 | 22.19 | 20.83 |
| **Pubertal onset, (%)** | 96.53 | 93.82 | 92.86 |
| Total caloric intake, (kcal/day) | 2201.69 | 2306.27 | 2252.19 |
| Total minutes of activity, (minutes/day) | 918.42 | 917.56 | 926.56 |

Means or percentages are presented for continuous or categorical variables, respectively

Bolded covariates included in the fully adjusted substituting models for sedentary bout frequency.

**Supplementary Table S6:** Overall Associations between Potential Confounders and Bout Duration for Moderate to Vigorous Physical Activity (MVPA):

|  | **Bout duration of MVPA** | | |
| --- | --- | --- | --- |
|  | **Tertile 1**  Median= 0  n=663 | **Tertile 2**  Median= 1.00  n=149 | **Tertile 3**  Median= 4.03  n=141 |
| **Maternal characteristics (at time of child’s birth)** | | | |
| Years of education, (years) | 10.91 | 10.93 | 11.36 |
| Age at childbirth, (years) | 26.49 | 25.99 | 26.38 |
| **Parity (≥ 2), (%)** | 61.54 | 64.43 | 53.90 |
| Marital Status (married), (%) | 70.44 | 68.46 | 71.63 |
| **Enrolled in calcium supplement study, (%)** | 28.21 | 23.49 | 29.79 |
| **Youth characteristics (at birth)** | | | |
| **Girls, (%)** | 54.75 | 56.38 | 39.01 |
| Gestational age, (weeks) | 38.76 | 38.62 | 38.94 |
| Mode of delivery (vaginal delivery), (%) | 62.44 | 59.73 | 64.54 |
| Breastfeeding duration, (months) | 8.35 | 7.05 | 7.98 |
| Age, (years) | 15.43 | 15.23 | 14.80 |
| **Youth characteristics (at follow-up visit)** | | | |
| Body mass index, (kg/m^2^) | 22.37 | 22.36 | 20.93 |
| **Pubertal onset, (%)** | 96.53 | 94.63 | 92.20 |
| Total caloric intake, (kcal/day) | 2201.69 | 2255.38 | 2317.09 |
| Total minutes of activity, (minutes/day) | 918.42 | 918.44 | 923.78 |

Means or percentages are presented for continuous or categorical variables, respectively

Bolded covariates included in the fully adjusted substituting models for sedentary bout duration.

**Supplementary Table S7:** Linear Mixed Models for Substituting Awake Sedentary Bouts^1^ with Higher Intensities and Cardiometabolic Risk Factors:

|  | | | **WC (cm) ^2^**  N= 530, # obs.= 923 | **SBP (mm Hg)**  N= 533, # obs.= 930 | **DBP (mm Hg)**  N= 532, # obs.= 924 | **Log glucose (mg/dL)**  N= 389, # obs.= 677 | **Log TG (mg/dL)**  N= 387, # obs.= 675 | **Log HDL-C (mg/dL)**  N= 386, # obs.= 673 | **Log insulin (μIU/mL)**  N= 386, # obs.= 670 | **Log HOMA-IR**  N= 387, # obs.= 672 |
| --- | --- | --- | --- | --- | --- | --- | --- | --- | --- | --- |
| Substituting sedentary bout duration with light activity (minutes/ day) | Crude model^3^ | β | 0.07 | 0.13* | 0.02 | 0.0021* | -0.0044 | 0.0006 | -0.0083* | -0.0054 |
|  |  | 95 % CI | (-0.02, 0.16) | (0.04, 0.22) | (-0.05, 0.08) | (0.0008, 0.0035) | (-0.0094, 0.0006) | (-0.0014, 0.0025) | (-0.0137, -0.0030) | (-0.0113, 0.0006) |
|  | Adjusted model^3^ | β | -0.01 | 0.03 | -0.05 | 0.0012 | -0.0033 | 0.0009 | -0.0065 | -0.0050 |
|  |  | 95 % CI | (-0.05, 0.03) | (-0.06, 0.13) | (-0.12, 0.02) | (-0.0002, 0.0026) | (-0.0084, 0.0020) | (-0.0011, 0.0029) | (-0.0120, -0.0009) | (-0.0112, 0.0011) |
| Substituting sedentary bout duration with MVPA activity  (minutes/ day) | Crude model^4^ | β | -0.30* | -0.06 | -0.08 | -0.0004 | -0.0104 | 0.0012 | -0.0110 | -0.0136 |
|  |  | 95 % CI | (-0.47, -0.12) | (-0.23, 0.10) | (-0.22, 0.05) | (-0.0029, 0.0021) | (-0.0194, -0.0015) | (-0.0022, 0.0047) | (-0.0207, -0.0014) | (-0.0241, -0.0030) |
|  | Adjusted model^4^ | β | -0.072 | -0.06 | -0.07 | -0.0005 | -0.0089 | 0.0021 | -0.0091 | -0.0103 |
|  |  | 95 % CI | (-0.145, 0.001) | (-0.22, 0.10) | (-0.20, 0.06) | (-0.0029, 0.0020) | (-0.0179, 0.0001) | (-0.0014, 0.0055) | (-0.0188, 0.0005) | (-0.0209, 0.0002) |
| Substituting sedentary bout frequency with light activity (count/day) | Crude model^5^ | β | 0.55 | 0.87* | 0.12 | 0.0154* | -0.0329 | 0.0047 | -0.0578* | -0.0376 |
|  |  | 95 % CI | (-0.08, 1.18) | (0.25, 1.50) | (-0.35, 0.59) | (0.0061, 0.0246) | (-0.0674, 0.0016) | (-0.0088, 0.0182) | (-0.0952, -0.0204) | (-0.0790, 0.0037) |
|  | Adjusted model^5^ | β | -0.11 | 0.23 | -0.35 | 0.0082 | -0.0236 | 0.0058 | -0.0420 | -0.0335 |
|  |  | 95 % CI | (-0.38, 0.16) | (-0.40, 0.87) | (-0.83, 0.13) | (-0.0015, 0.0180) | (-0.0596, 0.0125) | (-0.0081, 0.0198) | (-0.0808, -0.0033) | (-0.0761, 0.0091) |
| Substituting sedentary bout frequency with MVPA activity (count/day) | Crude model^6^ | β (SE) | -2.14* | -0.37 | -0.53 | -0.0013 | -0.0764 | 0.0130 | -0.0636 | -0.0805 |
|  |  | 95 % CI | (-3.35, -0.92) | (-1.53, 0.79) | (-1.47, 0.41) | (-0.0184, 0.0157) | (-0.1387, -0.0141) | (-0.0115, 0.0375) | (-0.1312, 0.0040) | (-0.1544, -0.0067) |
|  | Adjusted model^6^ | β | -0.54 | -0.31 | -0.40 | -0.0031 | -0.0654 | 0.0198 | -0.0488 | -0.0571 |
|  |  | 95 % CI | (-1.05, -0.03) | (-1.44, 0.82) | (-1.32, 0.52) | (-0.02, 0.0139) | (-0.1279, -0.0028) | (-0.0047, 0.0443) | (-0.1161, 0.0185) | (-0.1308, 0.0165) |

^1^ A bout was defined as 5 minutes of uninterrupted time performing a specific activity intensity. Within a bout, we allowed for up to 30-second of change in the physical activity intensity before terminating the bout.

^2^ Waist circumference models were additionally adjusted for body mass index

^3^ Crude model includes bout duration spent in sedentary and light physical activity, and adjusted model was adjusted for mother’s enrollment in the calcium intervention study, parity status, child’s age, sex, total time of physical activity, and pubertal onset.

^4^ Crude model includes bout duration spent in sedentary and MVPA physical activity, and adjusted model was adjusted for mother’s enrollment in the calcium intervention study, parity status, child’s age, sex, total time of physical activity, and pubertal onset

^5^ Crude model includes bout frequency spent in sedentary and light physical activity, and adjusted model was adjusted for mother’s enrollment in the calcium intervention study, parity status, child’s age, sex, total time of physical activity, and pubertal onset

^6^ Crude model includes bout frequency spent in sedentary and MVPA physical activity, and adjusted model was adjusted for mother’s enrollment in the calcium intervention study, parity status, child’s age, sex, total time of physical activity, and pubertal onset

* p < 0.00625

** p <0.0001

Abbreviations: WC: waist circumference; SBP: systolic blood pressure; DBP: diastolic blood pressure; TG: triglycerides; HDL-C: high density lipoprotein cholesterol; HOMA-IR: homeostatic model assessment of insulin resistance
